# Mission: Educational

**DOI:** 10.1289/ehp.112-a806

**Published:** 2004-10

**Authors:** John Manuel

The mission of the NIEHS is to reduce the burden of human illness and dysfunction from environmental causes by understanding the interrelationship between environmental factors, individual susceptibility, and age. The institute pursues this mission primarily through biomedical research, but in order for these findings to impact human health, they must be relayed to the public—and that includes students, from kindergarteners who are just beginning to learn about air, water, and plants, to college students preparing for careers as scientists.

“Most American schoolkids understand the need to ‘save the environment,’ [but] most do not understand the interaction between environment and human health,” says Marian Johnson-Thompson, director of education and biomedical research development at the NIEHS. “Given how much that interaction can affect them personally and the importance of an informed citizenry in supporting wise government policies, we need to be involved in environmental health science education.”

Education is a component of many activities taking place throughout the NIEHS. Indeed, says Liam O’Fallon, program analyst for the NIEHS science education and outreach grant programs, “Science education is really a part of all our jobs here at the institute.”

## Curriculum for Change

O’Fallon chairs the newly formed NIEHS Science Education Committee, which brings together the diverse educational activities throughout the institute and focuses on how these can better address student and teacher needs at the local and national level. One of the outcomes of this collaborative effort has been the development of a comprehensive NIEHS environmental health science education website (**http://www.niehs.nih.gov/science-education/**). The website provides access to an enormous amount of information on environmental health science, including homework resources and online activities for students, lesson resources and classroom activities for teachers, and presentation materials for scientists. The site also lists opportunities for professional development, summer research and job opportunities, and tours and visits of the campus. [For more information on the site, see “NIEHS Environmental Health Science Education, p. A805 this issue.]

The bulk of the institute’s education efforts are aimed at boosting environmental health science education in kindergarten through twelfth grade (K–12). Various studies, including the High School and Beyond survey of the National Center for Education Statistics, have cited a steady decline in both the scientific literacy of American students and the number of students interested in pursuing careers in natural science or engineering. Through its K–12 educational programs, the NIEHS seeks to help reverse both these trends.

Starting in 1994, the institute provided grants to universities to develop K–12 level instructional materials on such topics as cell biology, toxicology, risk assessment, scientific process and methodology, and indoor and outdoor air pollution. These instructional materials were meant to be incorporated into existing curricula.

The institute followed these instructional materials development grants with grants aimed at teacher enhancement and development. Grantees provided teachers with materials and curricula pertaining to environmental health science, funding to attend workshops, and opportunities to interact with environmental health scientists in the field. Under this initiative, grantees trained more than 7,500 classroom teachers to incorporate environmental health science into their classrooms. Some curricula, such as the My Health My World series produced by the Baylor College of Medicine for grades 2–4, have been so successful that they are being promoted nationally.

The latest initiative, Environmental Health Sciences as an Integrative Context for Learning (EHSIC), is intended to improve overall academic performance as well as enhance students’ comprehension of and interest in environmental sciences. These grants, which offer up to $250,000 per year for seven years, support projects designed to integrate environmental health science into a variety of school curricula. The nine recipients, several of whom received earlier grants for instructional materials and teacher development, are now entering the fifth year of their projects and are showing impressive results.

## Reaching the Grassroots

The University of Rochester Medical Center has used its EHSIC grant to develop multidisciplinary curriculum units for grades 5–12. All units have a problem-based learning component, include hands-on activities, and integrate science with other subjects such as health, English, and social studies. Kim LaCelle, formerly a science teacher at Marion High School in western New York and now a science educator at the University of Rochester Life Science Learning Center, describes activities that the students in the rural community of Marion found particularly relevant.

“We addressed local environmental health issues, such as how farmers handle agricultural waste,” LaCelle says. “With the NIEHS grant, we bought equipment to test wells for fecal coliforms. Another group mapped out the waterways that collected runoff from the fields and tested those for pesticides. The kids really enjoyed designing their own experiments. They developed a lot of confidence in their ability to do science.”

Cathy Hoppe, a special education teacher working with schools in west Rochester, found the activities well suited to her learning-disabled students. “The problem-based learning unit engaged my kids right from the start,” Hoppe says. “We presented them with a story about a child who discovers a polluted creek. They had to find out what kind of pollution it was. They used the Internet and went on field trips. It’s wonderful for them to be able to get out of the classroom and do field studies.”

In addition to its grant programs promoting environmental health science education in the schools, the NIEHS reaches the general population through its 25 NIEHS centers. The NIEHS requires each center to develop and maintain a Community Outreach and Education Program (COEP). Each center defines the community and/or region it serves and develops outreach efforts that are specific to the environmental health issues of greatest concern to that community.

For example, the COEP at the University of Texas Medical Branch (UTMB) offers a program called the Youth Environmental Studies Lab School, or YES! The program was designed to provide an intense, passionately taught, language-rich, small class environment to at-risk middle school children from the Galveston school system. At Central Middle School, all lessons in environmental science, math, reading, writing, and social studies coordinate around the environmental theme of the week. Students study the environment in a pattern of concentric circles: their own neighborhood, Galveston Island, the county, southeast Texas, and eventually, by extrapolation, the natural world. In another UTMB effort, the Bench Tutorials program pairs high school students with a university graduate student, postdoctoral fellow, or faculty mentor for supervised instruction and research in field study on the molecular biology of asthma.

“I feel our educational efforts through the schools have been very successful,” says Sharon Petronella, an assistant professor of pediatrics at UTMB.

“We don’t yet have a means of determining the impact of our educational programs on morbidity, but that may one day be possible. Last year, we conducted a survey of twenty thousand school kids in the Galveston area. The responses, including such information as number of days missed and reason for absence, may actually become a part of the students’ school health record. We will be able to see where the health problems are.”

Materials and resources developed by all 25 COEPs can be found at the COEP Resource Center (**http://www.apps.niehs.nih.gov/coeprc/**), a central repository of educational outreach materials produced by NIEHS grantees.

## Opening Doors to the Future

The NIEHS conducts a variety of science education programs in and around its Research Triangle Park, North Carolina, campus. Prominent among these are annual teacher workshops cosponsored with groups such as the North Carolina Association of Biomedical Research. During the one-day workshops, teachers hear from NIEHS researchers about the latest developments in toxicology research and visit the institute’s extensive lab facilities. They are provided with a curriculum titled Chemicals, the Environment, and You for use in the classroom. On average, the NIEHS sponsors two workshops per year attracting 40–50 teachers from the local area.

NIEHS also serves as a resource for programs at nearby universities and organizations that expose local high school and college students to possibilities for research and science careers. Students with, for example, the Research Apprenticeship Program developed by the University of North Carolina at Chapel Hill or Summer Ventures, a statewide program in which nearby North Carolina Central University participates, can visit the NIEHS campus, where they hear scientific presentations from institute staff, engage in informal discussions about career options and summer internship opportunities, and visit the laboratories.

Through its Summers of Discovery program, the NIEHS provides high school, college, and graduate-level students, science teachers, and college faculty with two-to three-month research internships in an NIEHS lab. Participants receive one-on-one mentoring with an institute scientist and attend weekly seminars where they discuss current research being conducted at the institute with the scientists in charge. At the end of the summer, students participate in a poster session at the NIEHS, where they make a brief oral presentation on their research and respond to questions as they would at a scientific society meeting. As a result of their internships, some students end up getting their names on peer-reviewed papers and/or being hired at the NIEHS.

## Lessons for Learning

According to Johnson-Thompson, statistics show that by the third grade, girls and minorities tend to lose interest in science because of cultural expectations that they pursue other careers, and minorities in particular don’t see any role models in science. One effort to break this trend is the Bridging Education, Science, and Training (BEST) Program, in which the NIEHS and the NIH partner with public schools in nearby Durham to nurture interest in environmental health science among economically disadvantaged students.

Through BEST, the institute provides schools with surplus supplies and equipment. Staff members give presentations at schools, and act as mentors and science fair judges. And the NIEHS supports science-based programs in the public schools and hosts Durham students in mini summer intern-ships, student research presentations, and awards programs.

Two of the schools in the BEST Program are C.C. Spaulding Elementary School and Shepard Magnet Middle School. C.C. Spaulding is designated as a Biosphere Magnet with a curriculum that has a strong focus on the environment. The school features a Life Lab Biostation containing several live ecosystems, which promotes scientific thinking and learning. Shepard, meanwhile, served as a pilot site for teaching the national Biological Sciences Curriculum Study science curriculum, which teaches science in the context of themes and issues relevant to the students themselves. Shepard currently is participating in Technology Enhanced Learning in Science, a National Science Foundation program that uses innovative, technology-enhanced curricula to teach scientific concepts and methods.

Another BEST experience points out what else is needed to successfully implement such programs. In 1996, the NIEHS worked with Durham’s Hillside High School to construct a Molecular Biology Laboratory and Training Center. The institute loaned the school $60,000 worth of state-of-the-art lab equipment, trained teachers in its use, mentored students, and provided judges for science contests. The center scored some notable successes early on, with several students winning area science competitions and performing summer internships at universities and corporations in Research Triangle Park.

But despite intensive financial and staff support from the NIEHS, the Hillside center has not proven to be a sustainable resource. According to Kenneth Cutler, former Hillside science teacher and now project director of the Berkeley, California–based Project SEED (Summer Educational Experience for the Disadvantaged), too few students had the skills and experience necessary to take advantage of the lab. Cutler offers some lessons about introducing science education programs to high school students.

“In order for students to take advantage of a sophisticated science laboratory, they need to be prepared in the fundamentals—mathematics, reading, writing,” Cutler says. “This needs to happen early, well before they reach high school. Students especially need to know how to write in order to communicate their findings and to make presentations. Students should be encouraged to take higher-level courses to prepare them for scientific thinking and methodology. And they should be provided with paid summer research internships to keep them involved and motivated. Finally, you’ve got to have support for the program at every educational level.”

## Free for Teachers

Besides the institute’s numerous funding opportunities, the NIEHS Office of Communications and Public Liaison produces educational booklets for use by school audiences and the general public. Students can use booklets such as *Environmental Diseases from A to Z* and *It’s Your Scene, Teen* for a variety of in-class activities. The office publishes eight brochures aimed at K–12 audiences, covering such topics as common environmental hazards, genetic predisposition, environment-related diseases, and air pollution. Teachers can request up to 60 copies of each publication for free by calling 919-541-3345 or e-mailing the NIEHS at booklet@niehs.nih.gov.

Along with the formal educational programs sponsored by the institute, individual staff members devote countless hours to education-related activities. By all accounts, NIEHS scientists enjoy the opportunity to get out of the lab and interact with the public. Perhaps more importantly, they also consider it their responsibility to play a role in guiding the next generation of environmental health scientists and ensuring that students evolve into scientifically literate citizens.

“It’s one of the more pleasurable things we do,” says NIEHS senior investigator Jerry Yakel. “Students get really jazzed up by the science, and some of them do, in fact, end up pursuing careers in the field.”

Over the last decade, science education activities at the NIEHS have positively impacted many lives across the nation, O’Fallon says. Through these activities, he says, students have won awards for academic performance in science, competed successfully for internships, and engaged in community-based activities aimed at improving local environmental conditions. Teachers have implemented engaging environmental health curricula in their classrooms. Communities have made policy changes aimed at improving the local environment. The result is a citizenry that better understands the connections between environment and health.

## Figures and Tables

**Figure:**
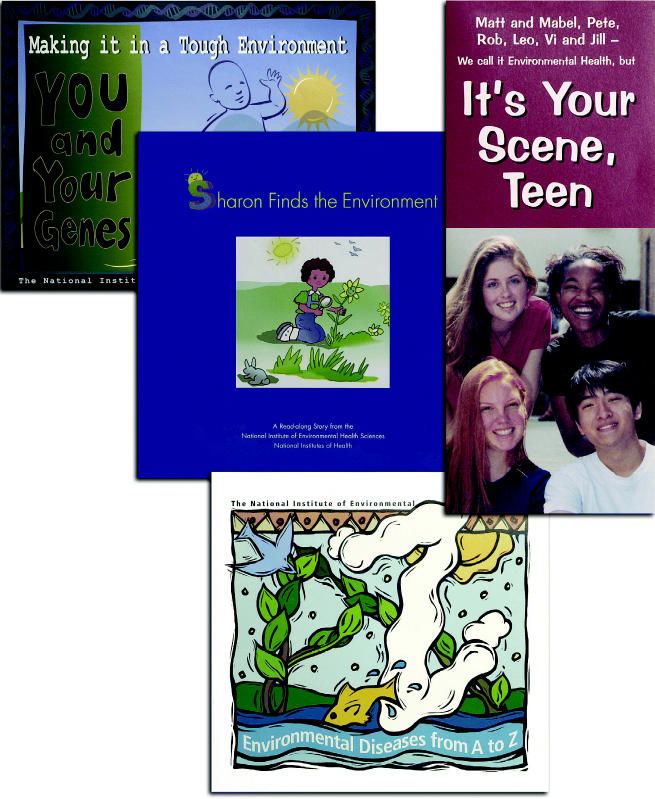
**Class-y materials.** The NIEHS produces a number of free environmental health educational materials for teachers to use in the classroom.

**Figure:**
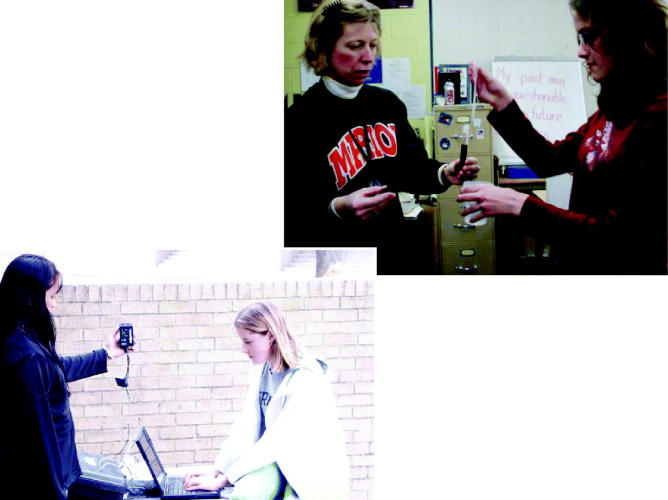
**Education abroad.** The NIEHS provides grants for environmental health education programs around the country. At Marion High School in New York (above), students test well water for fecal coliforms. The Bench Tutorials program in Galveston, Texas, (left) pairs high school students with graduate students, postdoctorate fellows, and faculty mentors to learn to conduct field studies of environmental toxicants such as air pollutants.

**Figure:**
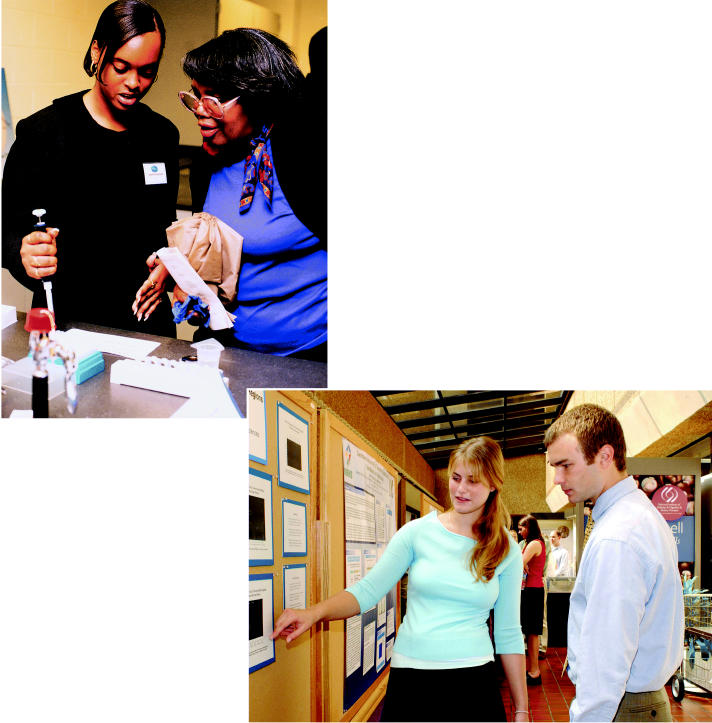
**Education at home.** The BEST Program provides equipment and supplies to local public schools (above left). Through the Summers of Discovery program, students and teachers are invited annually to train in NIEHS labs. At the end of the summer, participants present their own research projects (right).

